# Microstructure, Crystallographic Texture, and Mechanical Properties of Friction Stir Welded Mild Steel for Shipbuilding Applications

**DOI:** 10.3390/ma15082905

**Published:** 2022-04-15

**Authors:** Mohamed M. Z. Ahmed, Mohamed M. El-Sayed Seleman, Kamel Touileb, Ibrahim Albaijan, Mohamed I. A. Habba

**Affiliations:** 1 Mechanical Engineering Department, College of Engineering at Al-Kharj, Prince Sattam Bin Abdulaziz University, Al-Kharj 16273, Saudi Arabia; k.touileb@psau.edu.sa (K.T.); i.albaijan@psau.edu.sa (I.A.); 2 Department of Metallurgical and Materials Engineering, Faculty of Petroleum and Mining Engineering, Suez University, Suez 43512, Egypt; mohamed.elnagar@suezuniv.edu.eg; 3 Mechanical Department, Faculty of Technology & Education, Suez University, Suez 43518, Egypt; mohamed.atia@suezuniv.edu.eg

**Keywords:** mild steel, FSWed, microstructure, ferrite, bainite, crystallographic texture, mechanical properties, shipbuilding applications

## Abstract

In the current work, mild steel used in shipbuilding applications was friction-stir-welded (FSWed) with the aim of investigating the microstructure and mechanical properties of the FSWed joints. Mild steel of 5 mm thickness was friction-stir-welded at a constant tool rotation rate of 500 rpm and two different welding speeds of 20 mm/min and 50 mm/min and 3° tool tilt angle. The microstructure of the joints was investigated using optical and scanning electron microscopes. Additionally, the grain structure and crystallographic texture of the nugget (NG) zone of the FSWed joints was investigated using electron backscattering diffraction (EBSD). Furthermore, the mechanical properties were investigated using both tensile testing and hardness testing. The microstructure of the low-welding-speed joint was found to consist of fine-grain ferrite and bainite (acicular ferrite) with an average grain size of 3 µm, which indicates that the temperature experienced above A1, where a ferrite and austenite mixture is formed, and upon cooling, the austenite transformed into bainite. The joint produced using high welding speed resulted in a microstructure consisting mainly of polygonal ferrite and pearlite. This could be due to the temperature far below A1 experienced during FSW. In terms of joint efficiency expressed in terms of relative ultimate tensile, the stress of the joint to the base material was found to be around 92% for the low-speed joint and 83% for the high-welding-speed joint. A reduction in welding was attributed to the microstructure, as well as the microtunnel defect formed near the advancing side of the joint. The tensile strain was preserved at 18% for low welding speed and increased to 24% for the high welding speed. This can be attributed to the NG zone microstructural constituents. In terms of crystallographic texture, it is dominated by a simple shear texture, with increased intensity achieved by increasing the welding speed. In both joints, the hardness was found to be significantly increased in the NG zone of the joints, with a greater increase in the case of the low-welding-speed joint. This hardness increase is mainly attributed to the fine-grained structure formed after FSW.

## 1. Introduction

Ships are built, for the most part, by joining steel sheets in different configurations [1,2,3]. Welding is one of the most important techniques for joining steels with various joints and thus permits the development of expansive vessels utilizing completely welded joints owing to advancements in welding technology [4,5,6,7]. In the shipbuilding industry, gas metal arc welding (GMAW) and shielded metal arc welding (SMAW) are the most used welding methods [8,9,10,11]. However, the requirement for high-quality welds, the need to remove the formed slag after welding, cold cracking, and the distortion of the workpiece caused by traditional welding techniques have intensified the search for alternative welding processes. Friction stir welding (FSW) appears to be a better choice than the conventional welding processes, considering its advantages [12,13,14]. Since it was applied to joining aluminum (Al) alloys in 1991 and later developing welding tool materials, this technique has been used extensively in welding similar and dissimilar joint configurations of various steels [15,16,17,18,19,20]. For FSW of steel, new FSW tool designs and materials have been developed due to the high melting point of steel materials [3,21,22]. Much work has been done to weld different types of steels for various engineering applications via friction stir welding [3,23,24,25]. Cui et al. [26] examined the effect of FSW welding speeds from 25 to 400 mm/min at a constant rotational speed of 400 rpm on the 1.6 mm butt-joint properties of ultra-low-carbon (IF) steel and four different plain carbon sheets of steel (S12C, S20C, S35C, and S50C) using a tungsten carbide (WC) tool. The results correlated the slight enhancement in hardness and strength of the welded joints with the increase in welding travel speed. Ghosh et al. [27] friction-stir-welded 2 mm thick plain carbon steel in butt joints at travel speeds from 67 to 150 mm/min and rotation speeds from 710 to 1120 rpm, also using a WC tool. They concluded that the joint tensile strength decreased compared to the base material (BM), and grain growth in the heat-affected zone (HAZ) was detected under the applied parameters for all produced joints. Azevedo et al. [28] studied the FSW of 4 mm thick GL-A36 steel for the shipbuilding industry using two different tool materials: tantalum and polycrystalline cubic boron nitride (PCBN). The results confirmed enhancement in the nugget (NG) zone hardness, and the fatigue properties were close to the BM. Cater et al. [29] examined the distortion through 2 m lengths of DH36 and E36 marine steels welded by FSW and SMAW techniques. The results indicate lower distortion in the joints welded by the FSW process. Moreover, the tensile and fatigue properties outperformed the those achieve with the SMAW technique. Nathan et al. [30] reported significant hardness improvement in the weld zone for friction-stir-welded (FSWed) 5 mm marine grade HSLA steel (0.08% C and 1.42% Mn) compared to welded zones produced by SMAW and GMAW fusion welding techniques. This enhancement in hardness is ascribed to the dynamically recrystallized fine grain in the NG during FSW. Cui et al. [31] succeeded in producing FSWed defect-free 1.6 mm butt joints of high carbon steel S70C (0.72 wt.% C) and related the obtained microstructure and improved hardness to the thermal cycle during the welding process. Bhatia and Wattal [32] produced 3 mm butt-joint AISI 1018 steel under FSW conditions of 750 rpm and 60 mm/min using a WC tool. The results showed grain refining in pearlite and ferrite in the NG, and dendrite growth was also detected. Joint efficiency was 99.5%, with a reduction in elongation of 10% compared to the BM. Khodir et al. [33] noticed the presence of very fine pearlite and martensite structures in the NG of the 2 mm bead-on-plate welds of high carbon steel SK4 (0.95% C) FSWed at a constant 100 mm/min travel speed and rotation speeds of 200 and 400 rpm. Based on the above review, FSW has found its place among welding techniques in the field of shipbuilding. Despite the development of this industrial sector in Egypt, traditional welding techniques are still used. This work is considered the first attempt to introduce FSW technology to build the hull of ships from mild steel in the Suez Shipyard company in Suez, Egypt. Thus, in this work, we investigate the feasibility of using one-path FSW without pre- or post-heat treatment on shipbuilding mild steel plates to produce 5 mm butt joints at a constant rotation speed of 500 rpm and travel speeds of 20 and 50 mm/min. The mechanical properties of the welds were examined in terms of hardness and tensile properties. Moreover, the microstructure features were investigated with an optical microscope (OM) and a scanning electron microscope (SEM) equipped with an advanced electron backscattered diffraction (EBSD).

## 2. Materials and Methods

### 2.1. Material and FSW Parameters

**Figure 1:**
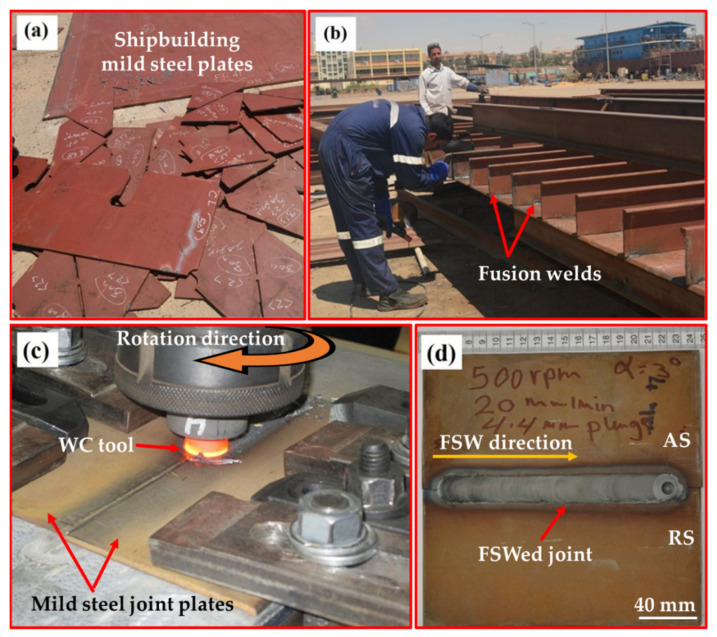
(**a**,**b**) Images of mild steel shipbuilding plates and conventional fusion welding during the manufacturing process, respectively. (**c**,**d**) Images of the FSW process of mild steel plates and a top view of the produced butt joint, respectively.

Mild LR-FH32 steel plates with dimensions of 100 mm width, 200 mm length, and 5 mm thickness supplied by Suez Shipyard Company in Suez, Egypt. were used for the FSW experiments. Table 1 and Table 2 represent the chemical composition and the tensile properties of the mild steel BM. FSW was conducted using a WC tool of dimensions 4 mm pin length, 6 mm pin diameter, and 20 mm shoulder diameter. The FSW parameters used were 500 rpm rotation rate and 20 and 50 mm/min traverse speeds. The tool tilt angle and the penetration depth were maintained at 3° and 4.4 mm, respectively, in all welds. FSW machines with 22 KW power, 3000 rpm max spindle speed, 1000 mm/min max welding speed, 100 KN max vertical force, and a tilting facility of ±5 degrees were used to carry out the welding process [34,35]. Figure 1 shows images of the mild steel plates used in shipbuilding (Figure 1a), conventional fusion welding in shipbuilding (Figure 1b), the FSW process of mild steel plates (Figure 1c), a the top view of an FSWed butt joint (Figure 1d). 

### 2.2. Microstructure Characterization

After FSW of the mild steel plates, the butt joints were sectioned perpendicular to the welding direction (WD) for subsequent characterization in terms of metallographic, hardness, and tensile testing. For macro- and microstructure investigations, the transverse cross-sections were prepared according to the standard metallographic procedures, starting with mechanical grinding with different grades of emery paper, followed by mechanical polishing using 0.05 µm alumina particles. Then, the samples were etched using nital of 2% Nitric acid in methanol. The optical microstructure was investigated using an Olympus optical microscope (OM), and a Quanta FEG 250 scanning electron microscope (Hillsboro, OR, USA) was used for SEM microstructure investigations. Furthermore, for the investigation of grain structure and crystallographic texture, the electron backscattered diffraction (EBSD) system within a Quanta FEG 250 SEM was used for the as-polished samples. A fully automated EDAX-EBSD system in a Quanta FEG 250 SEM equipped with a Hikari EDAX-EBSD camera EDAX-OIM7.3 (EDAX Inc., Mahwah, NJ, USA) was used for the acquisition of EBSD data; the system was controlled by orientation imaging microscopy data collection software EDAX-OIM7.3 (EDAX Inc., Mahwah, NJ, USA) operated at 20 kV. 

### 2.3. Characterization of Mechanical Properties 

For investigation of mechanical properties, both tensile testing and hardness testing were conducted. Tensile samples along the transverse direction of the FSWed butt joints were cut according to ASTM E8M-04. A computerized universal testing machine (Instron 4208, with a 300 kN load cell, Instron, Norwood, MA, USA) was used to conduct the tensile tests at a constant crosshead speed of 5 × 10^−2^ mm·s^−1^. A Vickers hardness tester (model HWDV-7S, TTS Unlimited, Osaka, Japan) with a load of 2 Kgf and a holding time of 15 s was used to evaluate the hardness. The hardness measurements in a grid a step of 0.5 mm in both the transverse direction (TD) and the normal direction (ND) of the joints covering the NG, as well as the thermomechanically affected zone (TMAZ) and the heat-affected zone (HAZ), were plotted in colored contour maps.

## 3. Results and Discussion

### 3.1. Top View and Transverse Section Macrographs of the FSWed Joints

**Figure 2:**
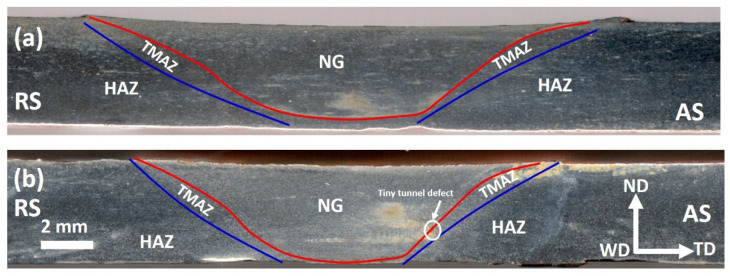
Transverse cross-section optical macrographs of the FSWed mild steel at a constant tool rotation rate of 500 rpm and welding speeds of (**a**) 20 mm/min and (**b**) 50 mm/min. TD: transverse direction, ND: normal direction, WD: welding direction.

The top view of the FSWed joint presented in Figure 1d shows a top surface free of any macro defects, with the stir zone having a grey color due to the removal of surface oxides during FSW. The transverse cross-section macrographs of the two joints are presented in Figure 2 with the different zones indicated, namely the NG zone in the center, the TMAZ, and the HAZ. The NG has a cone shape that widens towards the top surface, where the shoulder is affected and becomes narrow towards the base, where the tip of the pin is affected. It can be observed that the joint produced at a low welding speed (20 mm/min) is sound, without any macro defects. In contrast, the joint produced at the high welding speed of 50 mm/min has a tiny tunnel defect in the bottom of the NG at the interface between the NG and the TMAZ on the advancing side. This can be attributed to the cold condition of this joint, which resulted in high resistance of material for deformation and then defected joint consolidation. The formation of the internal tunnel defects is mainly explained by the lack of material flowability, which resulted in incomplete consolidation of the material behind the tool. During the FSW process, the material is taken in front the tool from the advancing side, moved to the retreating side, and then deposited behind the tool from the RS to the AS; thus, the last area of deposition is at the AS, and the tunnels are always formed at the AS. The main reason for the lack of flowability during FSW is high welding speeds, result in low temperature. 

### 3.2. Optical and SEM Microstructures 

**Figure 3:**
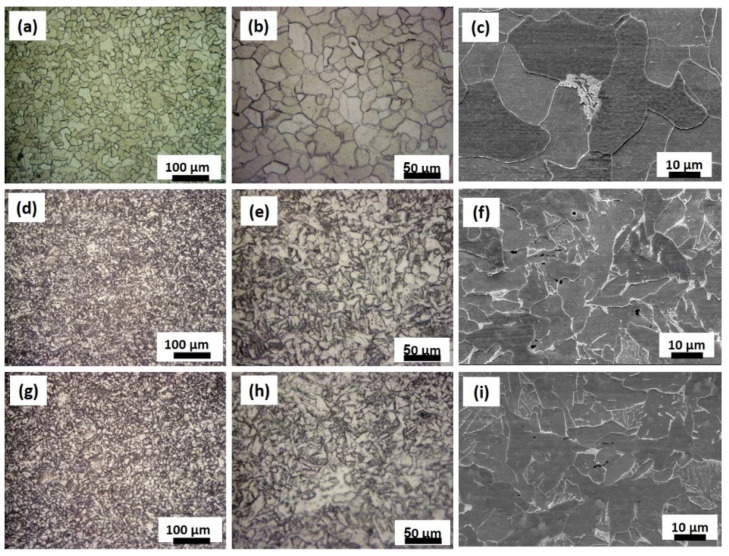
(**a**,**b**) OM and (**c**) SEM images of the mild steel BM microstructures, (**d**,**e**) OM and (**f**) SEM images of the FSWed joint produced at 20 mm/min near the top of the NG, and (**g**,**h**) OM and (**i**) SEM images near the base of the NG. (**b**,**e**,**h**) represent the higher magnifications of (**a**,**d**,**g**), respectively.

**Figure 4:**
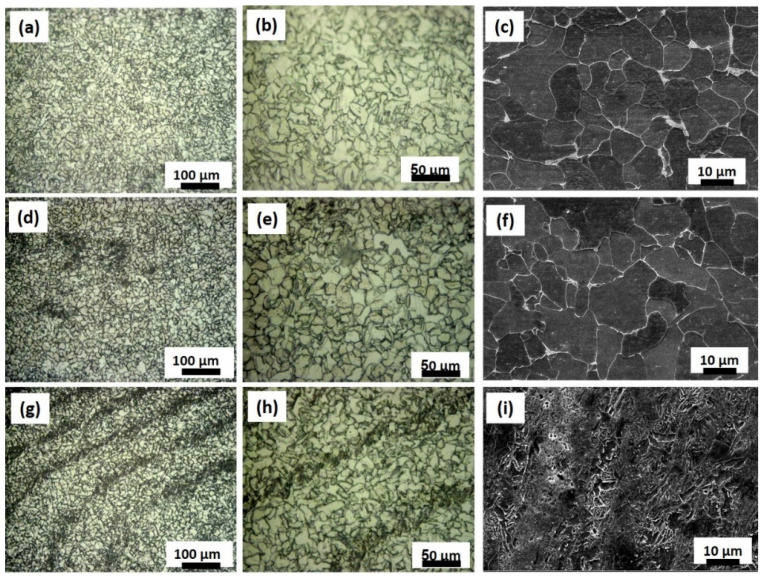
(**a**,**b**) OM and (**c**) SEM microstructure images near the top of the NG of the FSWed joint produced at 50 mm/min, (**d**,**e**) OM and (**f**) SEM images in the midsection of the NG, and (**g**,**h**) OM and (**i**) SEM images near the base of the NG. (**b**,**e**,**h**) represent the higher magnifications of (**a**,**d**,**g**), respectively.

The microstructure has been investigated using both optical microscope and scanning electron microscope in the NG zone and presented in Figure 3. Optical microstructure and SEM microstructure of the base material carbon steel (Figure 3a–c), FSWed joint produced at welding speed of 20mm/min near top of the NG (Figure 3d–f) and near base of the nugget (Figure 3g–i). The BM microstructure consists of coarse polygonal ferrite grains with some pearlite phases, as can be seen from Figure 3a–c. After FSW using a 20 mm/min welding speed, the microstructure in the NG zone was transformed into highly refined ferrite grains with a high density of fine acicular-shaped bainitic ferrites, with a similar microstructure near the top (Figure 3d–f) and near the bottom (Figure 3g–i) of the NG. Toumpis et al. [36] investigated the microstructure of FSWed carbon steel of 0.11 wt.% C for shipbuilding applications at a wide range of tool rotation rates in the range of 200–700 rpm and traverse speeds in the range of 100–500 mm/min. They reported that the coarse ferrite grains of the BM transformed into significantly refined ferrite grains and fine acicular-shaped bainitic grains at a welding speed of 120 mm/min and a tool rotation of 200 rpm. Furthermore, they reported that the bainite content was seen to increase with higher welding traverse speeds [36]. Additionally, Cui et al. [31] investigated the microstructure of FSWed 1.7 mm thick SAE-AIEI 1070 carbon steel plates in butt joints using rotation rates of 100–800 rpm and welding speeds of 25–400 mm/min. They reported that two methods could be used to control the microstructure of the FSWed carbon steel: either decrease the temperature upon FSW to below A1 or decrease the cooling rate to less than the lower critical cooling rate. As a result, a martensite microstructure is formed with a different percentage, in addition to a pearlite-ferrite microstructure at 400 rpm using all welding speeds. The martensite fraction increases by increasing the cooling rate, with a maximum of about 85 vol% at a 400 mm/min welding speed and a minimum of 16 vol% at a welding speed of 25 mm/min [31]. Compared with the FSW condition used in this work, i.e., 500 rpm and a speed of 20 mm/min, the carbon steel has lower carbon content (carbon equivalent ≈ 0.4). Thus, the obtained microstructure in this carbon steel mainly consists of refined ferrite grains and bainitic grains, taking into account the difference in the thickness (6 mm) and the carbon content. It is expected that the cooling rate in this case would be lower than the critical cooling rate that resulted in almost no martensite formed and that was replaced by a refined bainitic phase. A pearlite phase can also be observed in the NG zone. Increasing the welding speed to 50 mm/min at the same tool rotation rate of 500 rpm resulted in a microstructure that mainly consists of fine ferrite and pearlite, as can be observed in Figure 4a–i) which shows the optical and SEM microstructure near the top of the NG zone (Figure 4a–c), in the middle of the NG zone (Figure 4d–f), and near the bottom of the NG zone (Figure 4g–i). Increasing the welding speed results in a faster cooling rate relative to that experienced at a welding speed of 20 mm/min. However, it seems the cooling rate in this case is still far below the lower critical cooling rate that resulted in significantly refined ferrite and pearlite. Near the bottom of the NG, there is tinny tunnel (Figure 2b) that can be observed at the AS that resulted in a more refined microstructure with some onion rings c (Figure 4g–i). In order to further investigate this microstructural evolution, EBSD was carried out using different step sizes from 0.50 µm up to 0.25 µm, which will be presented and discussed in the next section. 

### 3.3. Grain Structure and Texture Using EBSD

**Figure 5:**
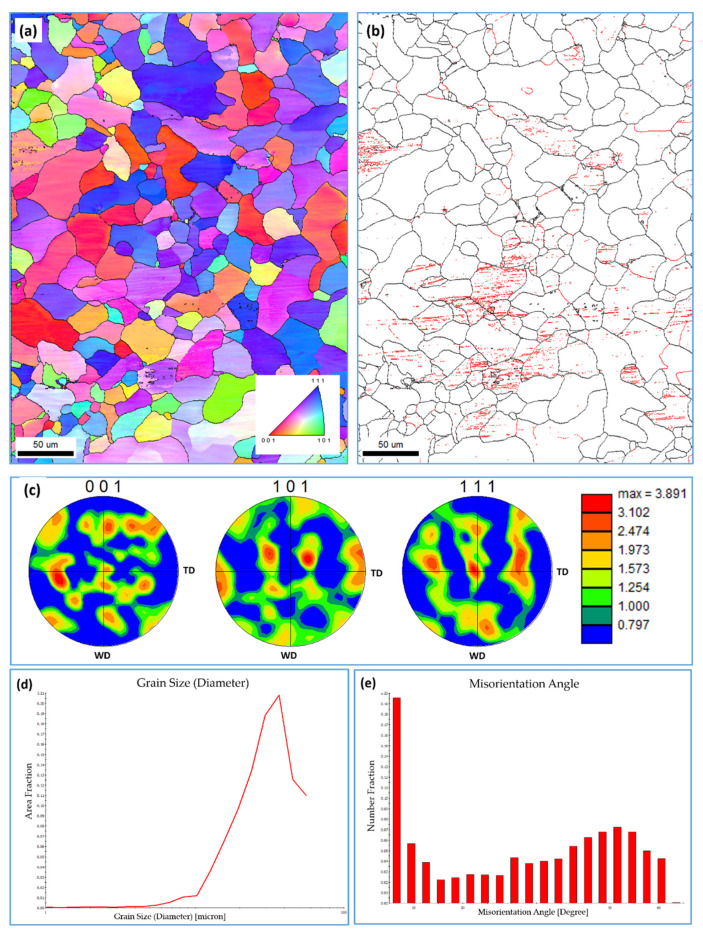
EBSD results obtained using a 0.5 µm step size of BM carbon steel. (**a**) IPF map with high-angle boundaries, (**b**) grain boundary map with low and high-angle boundaries, (**c**) 001, 101, and 111 PFs, (**d**) grain size distribution and (**e**) misorientation angle distribution.

BM mild steel was investigated using EBSD at a step size of 0.50 µm. Figure 5 shows the EBSD results of the BM mild steel; the inverse pole figure (IPF) map with high-angle boundaries (Figure 5a); the grain boundary map with low and high-angle boundaries (Figure 5b); 001, 101, and 111 PFs (Figure 5c); grain size distribution (Figure 5d); and misorientation angle distribution (Figure 5e). The BM microstructure mainly consists of equiaxed ferrite grains with an average grain size of about 15 µm. The grain size ranges from 2 µm up to 55 µm, with a high fraction towards coarse grain sizes, as can be observed in Figure 5d. The grain orientation is a mixture of red <001>, blue <111>, and green <101> orientations, as can be seen in Figure 5a. This indicates an almost weak texture, as can be seen from the pole figures in Figure 5c. The PFs show a weak rotated cube texture with only about four times random that is produced after hot rolling process. From the grain boundary maps shown in Figure 5b, it can be noted that almost the majority of the grains are free of low-angle boundaries, which indicates that dynamic recrystallization occurred upon hot rolling, with only some grains containing substructure, which could be due to the existence of a pearlite phase at those grains. Imam et al. [37] indicated that the low-angle boundaries inside ferrite grains are due to the existence of pearlite in the ferritic–pearlitic phase structure. The misorientation angle distribution is illustrated in Figure 5e, which indicates the dominance of the high-angle boundaries, with an appreciable fraction of low-angle boundaries. 

**Figure 6:**
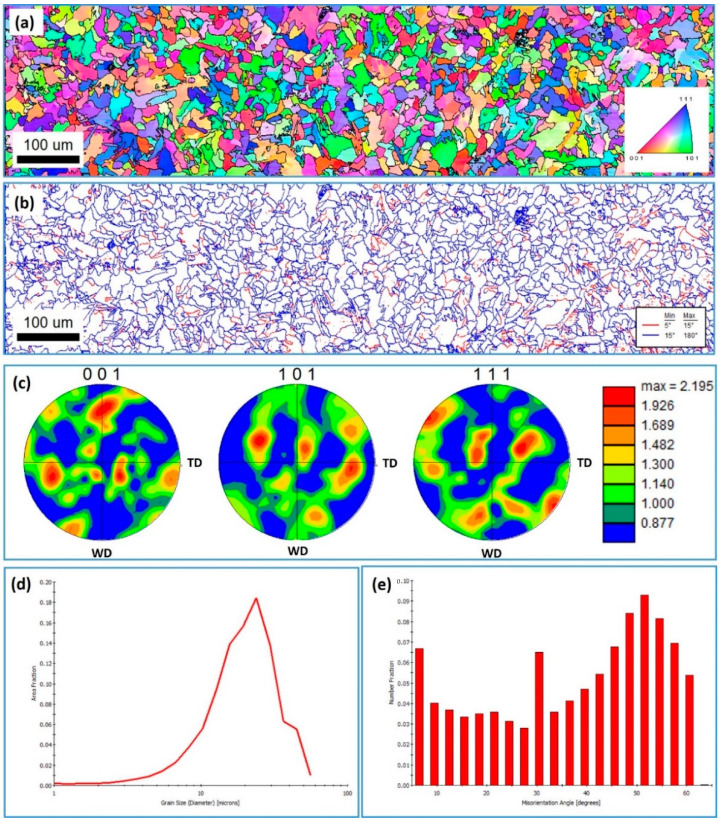
EBSD results obtained using a 0.5 µm step size at the NG zone of FSWed mild steel at 500 rpm and 20 mm/min welding speed. (**a**) IPF map with HABs > 15^o^ as black lines; (**b**) grain boundary map with 5° < LABs < 15° as red lines and HABs > 15° as blue lines; (**c**) 001, 101, and 111 PFs; (**d**) grain size distribution and (**e**) misorientation angle distribution at 20 mm/min.

**Figure 7:**
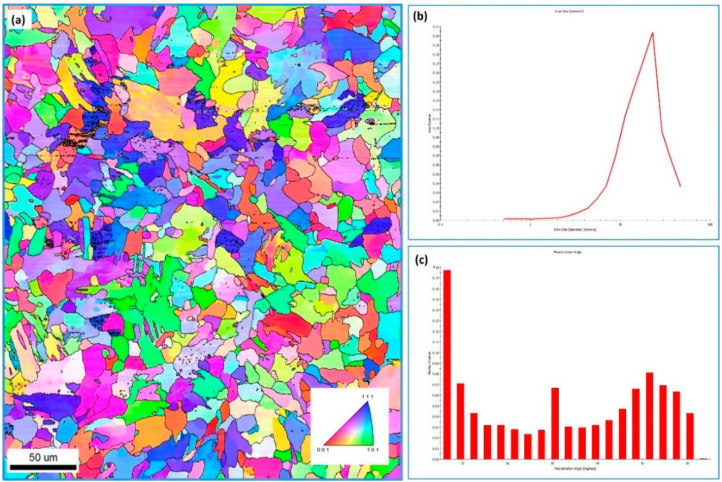
(**a**) High-resolution IPF coloring map acquired using a 0.25 µm step size in the NG of FSWed carbon steel at 20 mm/min, corresponding grain size distribution (**b**), and misorientation angle distribution (**c**).

The NG zone was also investigated using EBSD to examine the grain structure and texture evolution after FSW. Figure 6 shows EBSD results obtained using a 0.5 µm step size in the NG zone of FSWed mild steel at 500 rpm and 20 mm/min welding speed; the IPF map with high-angle boundaries (Figure 6a); the grain boundary map with low and high-angle boundaries (Figure 6b); 001, 101, and 111 PFs (Figure 6c); grain size distribution (Figure 6d); and misorientation angle distribution (Figure 6e). Additionally, Figure 7 shows a higher-resolution IPF map and corresponding grain sizes and misorientation angle distributions acquired using 0.25 µm for the NG of the same joint. The IPF maps in Figure 6a and Figure 7a indicate that the NG zone microstructure mainly consists of fine recrystallized ferrite with a high fraction of bainite (i.e., acicular ferrite) that seems to dominate the microstructure. The grain size distributions presented in Figure 6d and Figure 7c were obtained from the EBSD data acquired using 0.5 and 0.25 µm step sizes, respectively. It should be mentioned here that the step size affects the average grain size, as a reduction in step size results in a lower average grain size, as the use of a smaller step size captures smaller grains. Thus, the average grain size from the data collected using a 0.5 µm step size is 6 µm, and from those collected using a 0.25 µm step size, 3 µm. Imam et al. [37], in their study on the effect of online cooling on the microstructure of FSWed medium carbon steel, reported that the grain size could be reduced to less than 1 µm by rapid cooling. In the current study, the grain size was reduced to 3 µm without cooling by controlling the FSW parameters. Control of the grain size through the control FSW parameters was investigated by Sun et al. [38], Khodir et al. [33], and many others [26,31,39,40,41]. All reported the effectiveness of FSW parameter control in the achievement of fine-grained structure in carbon steel and the avoidance of martensitic transformations. The grain boundary map in Figure 6b presents the low-angle boundaries (LAB) from 5° to 15° as red lines and the high-angle boundaries (HAB) > 15° as blue lines. It can be observed that the microstructure is dominated by HABs, with only a few fractions of LABs. This can also be observed from the misorientation angle distribution presented in Figure 6e and Figure 7c. This low fraction of LABs indicates that the pearlite phase is minimal in this microstructure, as observed from the OM and SEM micrographs presented in Figure 2. Texture is presented as obtained data in 001, 101, and 111 PFs in Figure 6. In in the FSW process, the reference frame of the deformation process moves with the rotation of the tool in a circular path, as reported by Ahmed et al. [42,43,44]. Thus, for the texture to resemble the ideal positions, the EBSD data require rotations to align the deformation reference frame (shear deformation in this case; ϴ, z, r) with the sample reference frame (WD, TD, and ND). Although the texture is weak and only about three times random, it is a simple shear texture with the texture components away from their ideal positions due to the rotation of the shear reference frame in a circular path upon FSW. 

**Figure 8:**
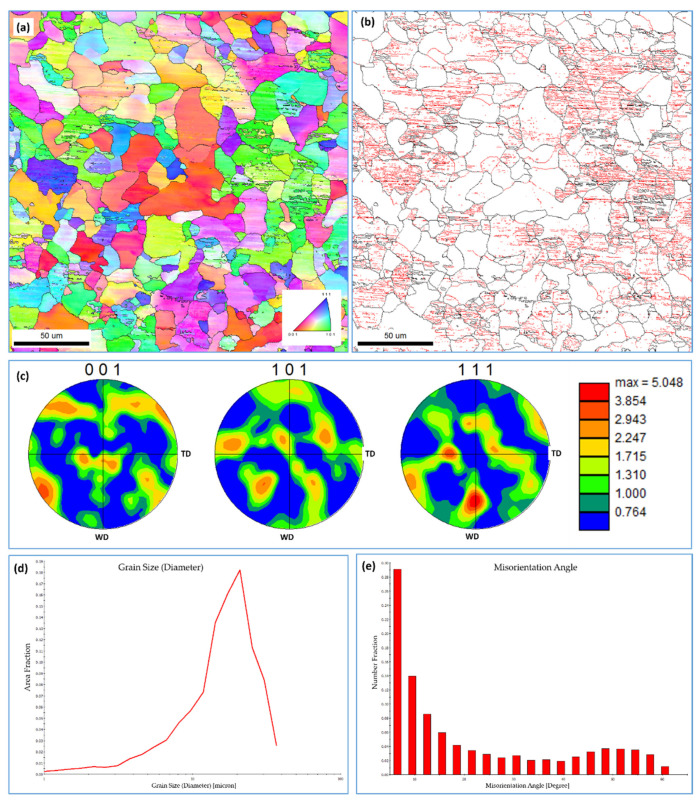
EBSD results obtained using a 0.5 µm step size in the NG zone of FSWed mild steel at 500 rpm and 50 mm/min welding speed. (**a**) IPF map with HABs > 15° as black lines; (**b**) grain boundary map with 5° < LABs < 15° and HABs > 15° as black lines; (**c**) 001, 101, and 111 PFs; (**d**) grain size distribution; and (**e**) misorientation angle distribution at 50 mm/min.

To examine the effect of the welding speed on the grain structure of the FSWed mild steel, the NG zone of the joint produced using a 50 mm/min welding speed was investigated using EBSD by acquiring OIM data with a 0.5 µm step size in the midsection of the NG. The results are presented in Figure 8: IPF map with HABs > 15° as black lines (Figure 8a); grain boundary map with 5° < LABs < 15° and HABs > 15° as black lines (Figure 8b); 001, 101, and 111 PFs (Figure 8c); grain size distribution (Figure 8d); and misorientation angle distribution (Figure 8e). The IPF map in Figure 8a clearly shows a mixed coarse and fine ferritic grain structure with a high density of pearlite that can be observed from the high density of LABs (Figure 8b). The average grain size obtained from the grain size distribution in Figure 8d is 4.7 µm, ranging from less than 2 µm up to 38 µm. It can be noted that the average grain size, in this case, is slightly lower than that obtained with the same step size for the joint welded at 20 mm/min. This can be attributed to the faster cooling rate due to the faster welding speed at the same rotation rate of 500 rpm. However, the major difference relative to the low-speed joint microstructure is the absence of the a bainitic grain structure (a circular ferrite), as the grain structure is mainly dominated by polygonal ferrite grains. This microstructure consisting mainly of polygonal ferrite and ferrite cementite (pearlite) structure indicates that the peak temperature in the NG zone is far below the A1 transformation temperature [37]. In terms of texture, the 001, 101, and 111 PFs in Figure 8c clearly indicate a simple shear texture with the texture components slightly away from their ideal position due to the rotation of the shear deformation reference frame. It can be noted that the texture is stronger than that obtained at a welding speed of 20 mm/min—more than five times random. This can be attributed to the faster cooling rate at the high welding speed, which allows for preservation of the deformation texture.

### 3.4. Mechanical Properties

**Figure 9:**
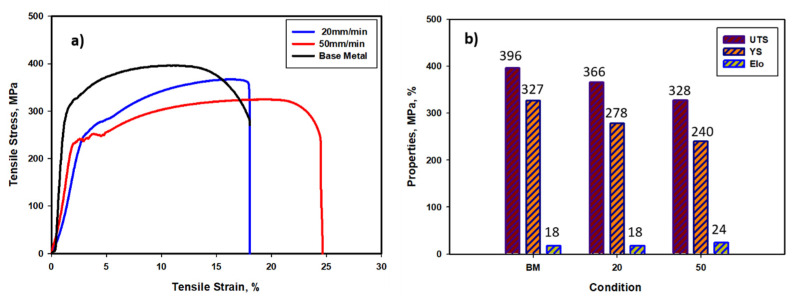
Stress–strain curves of the BM mild steel and the FSWed samples using 20 mm/min and 50mm/min welding speeds (**a**) and a comparison bar chart of the tensile properties (**b**).

To evaluate the mechanical properties of the FSWed mild steel joints, both a tensile test and a hardness measurement test were conducted along the transverse cross-section of the joints perpendicular to the welding direction. Figure 9 shows the stress–strain curves of the BM mild steel and the FSWed samples using 20 mm/min and 50 mm/min welding speeds in (Figure 9a) and a comparison bar chart of the tensile properties (Figure 9b). The tensile properties of the BM mild steel were reduced slightly after FSW, especially the UTS and YS. The UTS decreased from 396 MPa to 366 MPa after welding at a welding speed of 20 mm/min as was further reduced to 328 MPa by increasing the welding speed to 50 mm/min. The joint efficiency in terms of joint UTS relative to the BM UTS was 92% and 83% at welding speeds of 20 mm/min and 50 mm/min, respectively. The reduction in joint efficiency at a welding speed of 50 mm/min can be attributed to the tunnel defect observed at the AS of the joint, which represents crack initiation upon tensile testing. On the other hand, it can be noted that the E% was almost preserved at a welding speed of 20 mm/min at the level of 18% and increased to about 24% at a welding speed of 50 mm/min. This improvement in the E% can be attributed to the microstructural features obtained in each case. The ferritic bainitic structure allows for the preservation of tensile strain, whereas the polygonal ferrite microstructure allows for a significant increase in tensile strain across the transverse cross, even with the existence of the tiny tunnel defect. Toumpis et al. [36] investigated the joint efficiency of FSWed carbon steel for shipbuilding applications at rotation rates of 300, 200, and 700 rpm with combined welding speeds of 250, 100, and 500 mm/min, respectively. They reported that transverse tensile testing has consistently demonstrated that all slow and intermediate welds have higher YS than BM, whereas most fast weld samples fractured in the weld zone suggest reduced tolerance to parameter variations.

**Figure 10:**
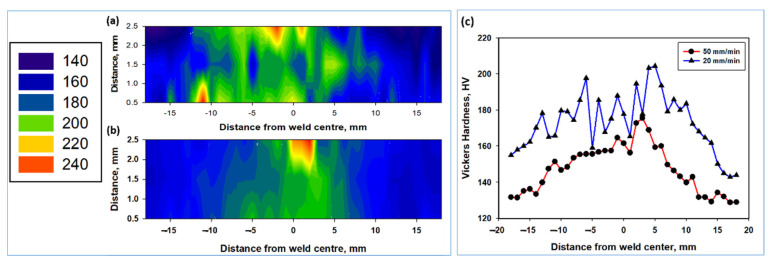
Color-contoured hardness maps across the transverse cross-sections obtained from the FSWed mild steel at a rotation rate of 500 rpm and welding speeds of (**a**) 20 mm/min and (**b**) 50 mm/min. Hardness curves along the transverse cross-sections in the midsection are presented in (**c**).

Hardness values are presented as hardness distribution color-contoured maps in Figure 10a,b and as comparison curves in Figure 10c. A noticeable increase in the hardness values can be observed in the NG zone in all cases, with an increase in the heat-affected zone width as a result of a reduction in the welding speed due to an increase in the heat input. This increase in the hardness of the NG zone can be attributed to the fine-grained ferritic and bainitic structure of the weld produced at a 20 mm/min welding speed. This also resulted in higher hardness values, as can be noted from the hardness curves in Figure 10c. The increase in the hardness of the weld produced at 50 mm/min is mainly attributed to the fine-grained polygonal structure, with average grain size of 4.7 µm. It can be noted that the microstructure consisting of ferrite and bainite resulted in higher hardness than that composed of fine polygonal ferrite and pearlite. This is mainly due to the characteristic features of the bainite, which consists of fine, very well dispersed carbide particles in the ferrite matrix, which make this phase (bainite) harder than the pearlite phase, resulting in a higher hardening effect. 

## 4. Conclusions

Based on the detailed investigation of the microstructural and mechanical properties of FSWed mild steel for shipbuilding applications, the following conclusions can be outlined:

1. The microstructure of the low-welding-speed (20 mm/min) joint consists of fine-grain ferrite and bainite (acicular ferrite) with an average grain size of 3 µm, indicating that the temperature experienced above A1, where a ferrite and austenite mixture is formed, and upon cooling, the austenite transformed into bainite. However, that of the high welding speed (50 mm/min), consisting mainly of polygonal ferrite and pearlite, indicated that the temperature experienced during FSW was below A1.
2. In terms of joint efficiency expressed in terms of relative ultimate tensile, the stress of the joint to the base material was found to be around 92% for the low-speed joint and 83% for the high-welding-speed joint.
3. Crystallographic texture is dominated by the simple shear texture, which is the typical texture of friction-stir-welded materials.
4. The hardness was increased in the NG zone of the joints, which is mainly attributed to the fine-grained structure formed after FSW.

## Figures and Tables

**Table 1:** Chemical composition of the mild steel (LR-FH32) BM used in shipbuilding applications.

Element	C	Si	Mn	Cr	Ni	Al	Cu	Ta	Nb	P	Fe
wt.%	0.154	0.205	1.330	0.062	0.061	0.044	0.012	0.037	0.019	0.008	Bal.

**Table 2:** Tensile properties of shipbuilding mild steel BM (LR-FH32).

Property	Ultimate Tensile Strength (UTS)	Yield Strength (YS)	Elongation (E%)	Fracture Strength (FS)
Value	396 MPa	327 MPa	18%	294 MPa

## Data Availability

Data will be available upon request through the corresponding author.
